# Sex-Related Differences in Outcomes for Oropharyngeal Squamous Cell Carcinoma by HPV Status

**DOI:** 10.1155/2022/4220434

**Published:** 2022-05-02

**Authors:** Derek D. Kao, Rocco M. Ferrandino, Deborah C. Marshall, Tinaye Mutetwa, Brett Miles, Joshua M. Bauml, Keith M. Sigel

**Affiliations:** ^1^Division of General Internal Medicine, Department of Medicine, Icahn School of Medicine at Mount Sinai, New York, NY, USA; ^2^Deparment of Otolaryngology, Icahn School of Medicine at Mount Sinai, New York, NY, USA; ^3^Department of Radiation, Icahn School of Medicine at Mount Sinai, New York, NY, USA; ^4^Division of Hematology/Oncology, Department of Medicine, Perelman School of Medicine, University of Pennsylvania, Philadelphia, PA, USA

## Abstract

**Background:**

Overall survival for HPV-associated oropharyngeal squamous cell carcinoma (OPSCC) has differed by sex, but little is known regarding cancer-specific outcomes. We assessed the independent association of sex with cancer-specific survival in patients with HPV-associated oropharyngeal squamous cell carcinoma (OPSCC).

**Methods:**

We identified 14,183 patients from the Surveillance, Epidemiology, and End Results (SEER) program with OPSCC and tumor HPV status. We used Kaplan–Meier methods to compare overall survival (OS) and OPSCC-specific survival (HNCSS) by patient sex and by tumor HPV status. We then separately fit multivariable survival and competing risk models evaluating the association of sex on these outcomes by tumor HPV status and stratified by the use of guideline-concordant OPSCC treatment.

**Results:**

A total of 10,210 persons with HPV-positive tumors (72.0%) and 3,973 with HPV-negative tumors (28.0%) were identified. A larger proportion of women had HPV-negative tumors (24.0%) versus HPV-positive tumors (13.2%; *p* < 0.001). Women with HPV-positive tumors were less likely to receive guideline-concordant treatment compared to men. In unadjusted survival analyses, women did not differ in OS or HNCSS compared to men for HPV-positive tumors but had worse OS and HNCSS for HPV-negative tumors. After adjustment, men and women with HPV-positive OPSCC did not differ in OS or HNCSS. However, women with HPV-negative tumors faced worse overall survival (hazard ratio (HR) 1.15, 95% CI 1.02–1.29) that persisted even after stratifying for stage-appropriate treatment (HR 1.28, 95% CI 1.11–1.47).

**Conclusions:**

Women with HPV-positive OPSCC had similar survival outcomes compared to men, but those with HPV-negative tumors have worse overall and cancer-specific survival.

## 1. Introduction

For the past 4 decades, the incidence of oropharyngeal squamous cell carcinoma (OPSCC) has steadily increased, growing by 122% in men and 43% in women from 1999 to 2015. [1–4] This rise is partially attributable to the increase in human papillomavirus (HPV) infection, the most common sexually transmitted infection among men and women in the United States. [5–8] OPSCC is now the most prevalent HPV-associated cancer. [1] Although not all OPSCC cases are associated with HPV infection, the proportion of HPV-related tumors among all OPSCC cases is increasing. Recent studies estimate that now over 70% of OPSCC cases are positive for p16, a biomarker frequently used to identify HPV-associated tumors. [9].

Incidence and temporal trends related to HPV-associated OPSCC specifically have also differed. Recent estimates have shown that HPV-associated OPSCC has increased annually 0.8% for women and 2.7% for men. [1] Although OPSCC disproportionately affects men and Whites, it appears to be increasing among all sex and race groups. [5, 10, 11].

OPSCC p16 status has been strongly associated with treatment responsiveness and survival outcomes. [12–16] However, outcomes of OPSCC in women, particularly those stratified by HPV status, have not been extensively studied and initial studies have conflicting conclusions regarding differences in survival. Although some studies have found that women have better overall survival with HPV-associated OPSCC, [17] others have found no difference. [18–20] Still others have found worse outcomes for women. [18, 19].

Previous OPSCC outcome studies have been limited by sample size or lack of data on cause of death, limiting the analysis to overall survival only, and, therefore, are susceptible to bias related to noncancer causes of death related to comorbid illnesses and behavioral factors. In addition, large cancer registry studies have lacked information on HPV status for OPSCC cases. In this study, we used data from a contemporary cohort of OPSCC cases from a national representative cancer registry database to compare overall and cancer-specific survival by sex, according to tumor HPV status. Our hypothesis is that women with OPSCC will have better survival outcomes regardless of HPV status, in line with trends seen in other cancers overall.

## 2. Materials and Methods

### 2.1. Patient Selection

We performed a retrospective cohort study using data from the Surveillance, Epidemiology, and End Results (SEER) Head and Neck with HPV Status Database. The SEER program has collected clinicopathologic data on incident cancer cases from 18 population-based registries, which represent approximately 28% of the US population. The Head and Neck with HPV Status Database includes cases diagnosed between 2010 and 2016, for which available HPV data have been collected. [21] Tumor HPV status in this database was determined through submission per the SEER Collaborative Stage Data Collection System, version 02.02–02.05 schemas. This included any testing performed on surgical specimens such as HPV in situ hybridization (ISH), tissue PCR, ISH for E6/7 RNA, real-time PCR for E6/7 RNA, and p16 immunohistochemistry. Blood or serology testing was excluded. HPV status was determined using the applicable collaborative stage site-specific factor 10 for each disease site schema throughout the data collection period. [22].

**Figure 1:**
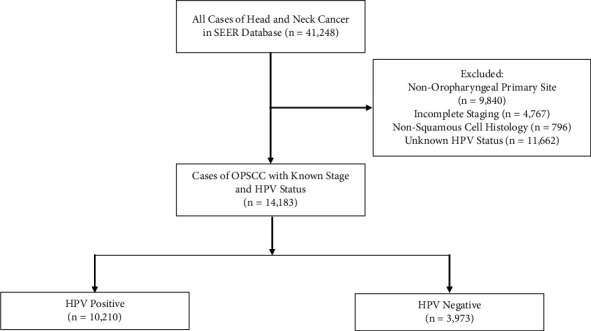
Included and excluded patients with oropharyngeal squamous cell carcinoma (OPSCC). SEER, Surveillance, Epidemiology, and End Results; HPV, human papillomavirus.

We identified incident cases of OPSCC using the *International Classification of Diseases for Oncology*, Third Edition (ICD-O-3), topography codes C01.9–10.9, which includes the base of the tongue, lingual tonsil, soft palate, uvula, tonsillar fossa, tonsillar pillar, tonsil, vallecula, anterior surface of the epiglottis, lateral and posterior wall of the oropharynx, branchial cleft, and oropharynx, not otherwise specified. Cases that were not of squamous cell histology, as determined by ICD-O-3 (8050–8084), were excluded. Next, we identified and included cases with known *American Joint Committee* on *Cancer*, 7th edition, clinical stage, which we restaged to the 8th edition based on available *T*, *N*, and *M* information on the individual level. Cases with unknown HPV status were excluded (*n* = 11,662). A total of 14,183 patients were identified (Figure 1). We then classified tumors according to SEER HPV data as HPV positive (*n* = 10,210) or HPV negative (*n* = 3,973).

### 2.2. Study Variables

**Figure 2:**
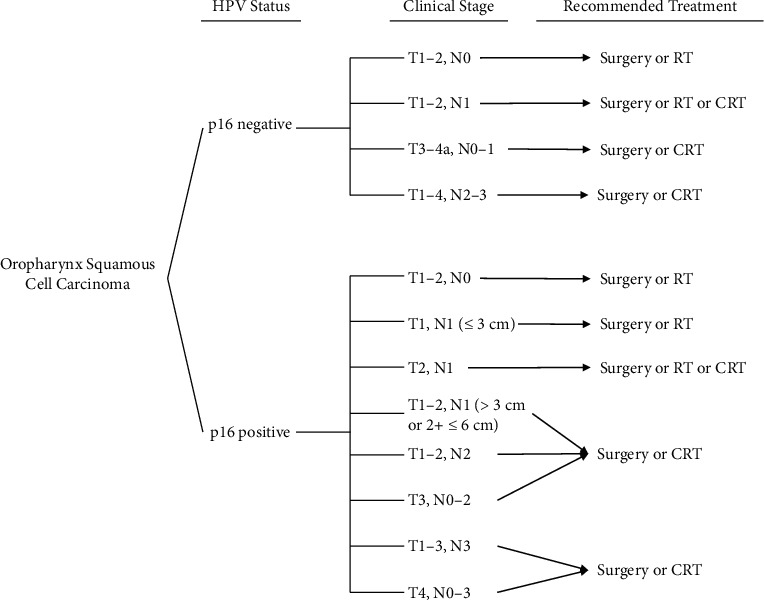
Summary of NCCN guidelines by HPV status and AJCC 8th edition clinical stage.

The SEER data were used to identify age, race/ethnicity, year of OPSCC diagnosis, median household income by county attribute, proportional educational attainment by county attribute, and insurance type (which we classified as uninsured, any Medicaid, insured, insured with no specifics, or unknown). Tumor characteristics (stage and *T*, *N*, and *M* values) and treatment type were ascertained from SEER on the individual level. We defined surgery as any definitive resection surgery performed, including excisional surgery of the oropharynx, the base of the tongue resection, palatectomy, uvulectomy, and/or tonsillectomy. Radiotherapy was coded for cases receiving beam radiation or a combination of a beam with implants or isotopes. Chemotherapy was included in the database and encoded as “yes” or “no/unknown.” Each case was then encoded with all treatments received, whether surgery, radiotherapy, or chemotherapy alone or in combination with other treatment methods. To determine whether the guideline-concordant treatment was administered by clinical stage, treatments were compared to *National ComprehensiveCancer* Network (NCCN) guidelines. [23] NCCN guidelines differ based on p16 status and clinical staging. An overview of NCCN guidelines can be found in Figure 2. To accommodate the categorical nature of the data available, we summarized the NCCN guidelines to whether surgery, chemotherapy, radiotherapy, or a combination of the three was recommended for each patient's p16 status and clinical stage. Treatment for each case was considered to meet recommended guidelines if the patient received at least the recommended modality of treatment.

The primary outcome in this study was head and neck cancer-specific death. Any cause of death due to cancer in head and neck structures was encoded as a head and neck cancer-specific death. Our secondary outcome was death from any cause. Survival times for primary analyses were calculated from months of survival provided in SEER data, defined as the time from date of diagnosis to either death or end of follow-up.

### 2.3. Statistical Analysis

We tested for differences in baseline and clinical characteristics between women and men stratified by tumor HPV status using the *t*-test for normal continuous variables and the chi-square test for categorical variables. We then compared the use of cancer treatments and stage-specific guideline-concordant treatments by sex, also stratified by tumor HPV status, using the chi-square test. To further evaluate differences in cancer treatment by sex, we fit multivariable models assessing sex as a predictor of guideline-concordant treatment, adjusting for age, race, educational attainment and income by county attribute, insurance type, cancer stage, and marital status. We then fit unadjusted Cox regression models to compare overall and cancer-specific survival by sex. Adjusted models of overall and cancer-specific survival were then run to measure the association of sex with these outcomes, separately adjusting for race, sociodemographic factors (median county household income, proportional county education attainment, and insurance type), cancer stage, and treatment by tumor HPV status and stratified by the use of guideline-concordant OPSCC treatment. We separately compared outcomes for all HPV-positive and HPV-negative OPSCC cases, then, stratified by tumor stage, and then, further stratified by use of guideline-concordant treatment, using the aforementioned models. We also tested for interactions between sex and tumor stage. Last, to evaluate the effects of potential differences in competing (non-OPSCC-related) causes of death by sex, we fit similar multivariable Fine-Gray regression models, stratified by HPV tumor status, first for all cases, and then limited to patients receiving guideline-concordant treatment. Complete models are provided in Supplementary Tables. All analyses were performed in STATA Version 13 (STATA Corp., College Station, TX). STATA code for all analyses can be found online at this link: https://github.com/derkao/opscc-by-sex. The Mount Sinai Institutional Review Board determined that this study was exempt from human research.

## 3. Results

### 3.1. Baseline Characteristics

In total, 14,183 cases of OPSCC with known staging and HPV status were identified, of which 10,210 (72.0%) were HPV positive and 3,973 (28.0%) were HPV negative (Table 1). Women were more likely to have HPV-negative tumors than HPV-positive tumors (24.0% versus 13.2%; *p* < 0.001). For HPV-positive OPSCCs, in comparison to men, women were older (*p* < 0.001), more likely to be non-Caucasian (*p*=0.001), early stage (*p* < 0.001), single (*p* < 0.001), and more likely to be insured with Medicaid (*p* < 0.001). Among HPV-negative OPSCC cases, women were also older compared to men at diagnosis (*p* < 0.001), earlier stage (*p* < 0.001), and more likely to be single (*p* < 0.001).

### 3.2. Treatment by Stage

In univariate analyses, women with HPV-positive OPSCC were significantly less likely to receive guideline-concordant treatment (Table 2; *p*=0.002) compared to men. For HPV-negative tumors, a disparity of similar magnitude was seen; however, the comparison was not significant (*p*=0.06). Differences in the use of guideline-concordant treatment for women with HPV-positive tumors appear to stem from less aggressive treatment for earlier-stage (I-III) cancers. In contrast, for HPV-negative tumors, women were less likely to receive guideline-concordant treatment for more advanced-stage cancers (stage III, *p*=0.03, stage IV, *p*=0.005). After adjusting for sociodemographic factors, including race, median county household income, proportional county education attainment, insurance type, cancer stage, and marital status, sex was still a significant predictor of guideline-concordant treatment for HPV-positive tumors (*p*=0.04; results not otherwise shown) but not HPV-negative tumors (*p*=0.09).

### 3.3. Survival Outcomes

In unadjusted survival analyses (results not otherwise shown for unadjusted survival analyses), men and women with HPV-positive tumors did not differ in overall survival (OS) (HR 1.05, 95% CI 0.90–1.22) or head and neck cancer-specific survival (HNCSS) (HR 1.17, 95% CI 0.95–1.44), defined by death due to cancer in any head and neck structure. However, in HPV-negative cases, women had worse OS (HR 1.27, 95% CI 1.13–1.43) and HNCSS (HR 1.29, 95% CI 1.10–1.51). Worse cancer-specific survival persisted for women after accounting for possible competing risks (HR 1.25, 95% CI 1.07–1.47). Among patients who received guideline-concordant treatment, we found similar trends; women had no difference in outcomes for HPV-positive tumors but had significantly worse HNCSS for HPV-negative tumors even after accounting for competing risks (*p* = 0.02).

**Figure 3:**
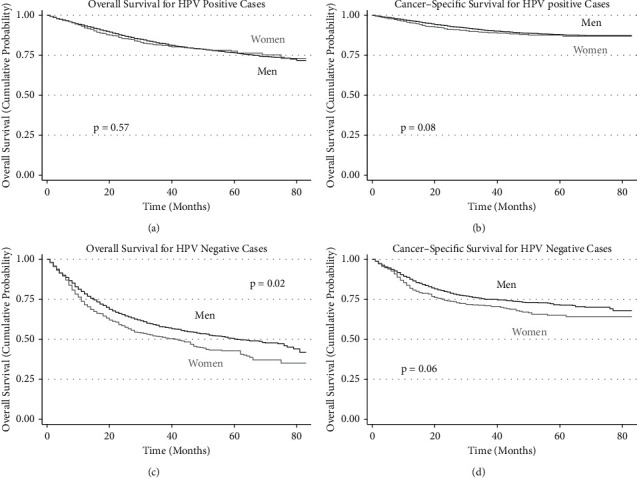
Kaplan–Meier curves. (a) Overall survival (OS) in HPV-positive cases. (b) Head and neck cancer-specific survival (HNCSS) in HPV-positive cases. (c) Overall survival (OS) in HPV-negative cases. (d) Head and neck cancer-specific survival (HNCSS) in HPV-negative cases.

In adjusted analyses, men and women had similar overall survival with HPV-positive tumors overall and among the subset of cases that received guideline-concordant treatment (Table 3, Figure 3(a)). This was true for head and neck cancer-specific survival as well (Table 3, Figure 3(b)). In multivariable competing risk models, HNCSS was also not different by sex after adjustment for all HPV-positive cases or among those receiving guideline-concordant treatment.

Survival differences between women and men were seen with HPV-negative tumors. Adjusted survival analyses for the overall HPV-negative tumor cohort showed decreased OS for women (HR 1.15, 95% CI 1.02–1.29, Figure 3(c)). This effect was larger in the subset of cases that received guideline-concordant treatment (HR 1.28, 95% CI 1.11–1.47). Statistically significant differences were also seen in stage III with guideline-concordant treatment, stage IVA overall, and stage IVB with guideline-concordant treatment. In HNCSS analysis, survival differences were not statistically significant for the overall cohort of HPV-negative tumors (Figure 3(d)). However, HNCSS was worse in women receiving guideline-concordant treatment in all stages combined (HR 1.30, 95% CI 1.07–1.59); this persisted after accounting for competing risks (HR 1.26, 95% CI 1.03–1.55). These differences were most prominent among patients with stage IVA and IVB tumors. In the analyses testing for statistical interactions between patient sex and tumor stage, there were no statistically significant interactions in groups where there were underlying differences in outcomes (OS or HNCSS) by sex.

## 4. Discussion

In this population-based cohort study of HPV-associated OPSCC, we found no differences in survival outcomes by sex for HPV-positive tumors but worse survival for women for HPV-negative tumors. This provides evidence that contrasts previous findings that women may have a worse prognosis with HPV-associated OPSCC. For HPV-negative tumors, we found evidence of treatment disparities by sex, and survival differences were still pronounced in women who received guideline-concordant treatment. Treatment-related factors such as tolerability or responsiveness or possibly tumor behavior may, therefore, differ for women with HPV-negative OPSCC. Other factors may include preemptive surgery and the timing of treatment, as early surgery of preneoplastic lesions has been shown to be beneficial. [24].

We found differences in treatment patterns for women and men with OPSCC. For both HPV-positive and HPV-negative tumors, women were less likely to receive guideline-concordant treatment. A recent large cohort study (*n* = 884) compared to head and neck cancer treatment by sex and found that women received less intensive treatment, despite worse cancer-specific survival. [25] However, this study did not examine differences by HPV status. Additionally, a matched-pair analysis of 572 female and male patients, in which treatment was a matched variable, demonstrated no significant differences in survival outcomes between men and women. [26] There are few studies that have examined treatment differences between men and women stratified by HPV status. In an analysis of the *National Cancer* Database, patterns of treatment for HPV-positive and HPV-negative OPSCC differed for women versus men; however, proportions of untreated patients were similar. [19] It is, therefore, likely that women experience cancer treatment disparities compared to men for OPSCC, similar to what is observed for other tumor types. [27–31] These disparities may be partially explained by a greater burden of comorbidities seen in women versus men with OPSCC, as demonstrated in population-based data. [32].

Prior comparisons of differences in prognosis for OPSCC by sex, particularly for those known to be HPV-associated, have been conflicting. In a two-site retrospective analysis of 239 cases, Yin et al. found that women with HPV-positive OPSCC had improved overall survival compared to men. [17] In contrast, three population-based studies have investigated overall survival by sex and tumor HPV status for OPSCC : Faraji et al. conducted a retrospective analysis using the *National Cancer* Database with cases from 2010 to 2015, Li et al. used the same database with cases from 2010 to 2014, and Razzaghi et al. used the *National Program of Cancer* Registries from the CDC. [18–20] These larger, population-based studies all found overall survival trends consistent with our study for HPV-positive tumors: women with HPV-positive tumors had no difference compared to men.

Differing from our findings, in HPV-negative OPSCC Yin et al. found no difference in overall survival between men and women using two-site retrospective data. Studies from population-based cancer registry data, however, found that women fared worse than men, in agreement with this study. [17–19] No previous study has had cause-of-death information, and however, our study shows that OPSCC-specific survival is no different for men and women for HPV-positive tumors but that differences in overall survival for HPV-negative tumors are partly driven by worse cancer-specific survival.

To our knowledge, this is the first study to examine survival between treatment modalities stratified by HPV status and sex. Feinstein et al. performed a retrospective analysis at a single regional veterans' health center and found no difference in survival between treatment modalities in all OPSCC cases and HPV-positive OPSCC cases alone. [33] A systematic review from 2015 also demonstrated no statistically significant difference in hazard ratios between patients treated with primary surgery vs. primary radiation. [34] Both studies found better survival in HPV-positive tumors, but neither of these studies looked at sex-based differences. These differences highlight the importance of patient counseling and increased awareness among providers regarding sex-related differences in outcomes of patients undergoing treatment for OPSCC.

### 4.1. Limitations

Strengths of this study include the population-based national dataset that provided a large sample size and heterogeneity of the patient population, enabling generalization to the U.S. population. This database also provided HPV status data that were determined by robust methods. However, our findings must be viewed in light of several limitations. First, the lack of comorbidity data limits the interpretation of our data. Of note, cancer-specific survival data were available and were used in this study. Second, detailed treatment information on chemotherapy and radiotherapy is not available; thus, we cannot interpret our data with respect to predicting response to treatment. Third, our dataset did not include information on behavioral (e.g., smoking) or environmental exposures, which may have impacted cancer outcomes. Fourth, missing HPV data may be a source of selection bias, as previous studies have demonstrated disparities of HPV testing by demographics such as race and insurance. [35] Lastly, this study utilized a relatively large number of statistical tests; therefore, some *p* values that were below the threshold for significance may represent results that occurred at random and not truly significant associations.

## 5. Conclusions

In this study of population-based cancer data, we found that outcomes for OPSCC did not differ for women compared to men for HPV-positive tumors. Women, however, did experience worse overall and cancer-specific survival for HPV-negative tumors, which was not fully explained by treatment disparities.

## Figures and Tables

**Table 1:** Baseline cohort characteristics by sex.

	HPV positive			HPV negative		
Characteristic	Men *N* = 8,860	Women *N* = 1,350	*p* value	Men *N* = 3,020	Women *N* = 953	*p* value
Age category, median (IQR)	60–64 (50–69)	60–64 (55–69)	<0.001	60–64 (55–69)	60–64 (55–74)	<0.001

Race/ethnicity, N (%)						
Caucasian	7,613 (85.9)	1,103 (81.7)	0.001	2,270 (75.2)	731 (76.7)	0.06
African American	454 (5.1)	98 (7.3)		394 (13.1)	118 (12.4)	
Hispanic	509 (5.7)	93 (6.9)		223 (7.4)	52 (5.5)	
Asian or Pacific Islander	200 (2.3)	43 (3.2)		104 (3.4)	46 (4.8)	
Other/unknown	84 (1.0)	13 (1.0)		29 (1.0)	6 (0.6)	

Year of diagnosis, N (%)						
2010	445 (5.0)	64 (4.7)	0.90	227 (7.5)	58 (6.1)	0.10
2011	676 (7.6)	114 (8.4)		358 (11.9)	125 (13.1)	
2012	986 (11.1)	158 (11.7)		414 (13.7)	126 (13.2)	
2013	1,312 (14.8)	205 (15.2)		479 (15.9)	140 (14.7)	
2014	1,575 (17.8)	238 (17.6)		519 (17.2)	153 (16.1)	
2015	1,777 (20.1)	260 (19.3)		464 (15.4)	182 (19.1)	
2016	2,089 (23.6)	311 (23.0)		559 (18.5)	169 (17.7)	

Stage at diagnosis, N (%)						
I	5,098 (57.5)	878 (65.0)	<0.001	214 (7.1)	121 (12.7)	<0.001
II	2,097 (23.7)	255 (18.9)		250 (8.3)	101 (10.6)	
III	1,451 (16.4)	181 (13.4)		563 (18.6)	170 (17.8)	
IV	214 (2.4)	36 (2.7)		—	—	
IVA	—	—		1,517 (50.2)	427 (44.8)	
IVB	—	—		296 (9.8)	77 (8.1)	
IVC	—	—		180 (6.0)	57 (6.0)	

Clinical T∗, N (%)						
0	61 (0.7)	6 (0.4)	<0.001	5 (0.2)	4 (0.4)	0.004
1	2,504 (28.3)	470 (34.8)		649 (21.5)	251 (26.3)	
2	3,560 (40.2)	542 (40.2)		1,004 (33.3)	284 (29.8)	
3	1,525 (17.2)	165 (12.2)		662 (21.9)	181 (19.0)	
4	1,210 (13.7)	167 (12.4)		—	—	
4a	—	—		467 (15.5)	157 (16.5)	
4b	—	—		174 (5.8)	66 (6.9)	
Missing	—	—		59 (2.0)	10 (1.0)	

Clinical N^†^, N (%)						
0	1,178 (13.3)	247 (18.3)	<0.001	746 (24.7)	325 (34.1)	<0.001
1	5,751 (64.9)	871 (64.5)		533 (17.7)	166 (17.4)	
2	1,534 (17.3)	198 (14.7)		31 (1.0)	10 (1.1)	
2a	—	—		217 (7.2)	57 (6.0)	
2b	—	—		797 (26.4)	216 (22.7)	
2c	—	—		468 (15.5)	140 (14.7)	
3	397 (4.5)	34 (2.5)		177 (5.9)	29 (3.0)	
Missing	—	—		51 (1.7)	10 (1.0)	
Median county Household income, $	63,880	63,250	0.10	61,020	61,020	0.45
% County education, median						
<9th grade	5.18	5.29	0.97	5.26	4.88	0.13
< High school	12.10	12.32	0.52	12.50	12.45	0.05
≥ Bachelors	32.59	31.93	0.35	31.23	31.23	0.33

Insurance, N (%)						
Uninsured	239 (2.7)	26 (1.9)	<0.001	132 (4.4)	36 (3.8)	0.41
Any Medicaid	783 (8.8)	158 (11.7)		521 (17.3)	182 (19.1)	
Insured	6,702 (75.6)	960 (71.1)		1,871 (62.0)	567 (59.5)	
Insured (no specifics)	1,036 (11.7)	184 (13.6)		441 (14.6)	153 (16.1)	
Unknown	100 (1.1)	22 (1.6)		55 (1.8)	15 (1.6)	

Marital status, N (%)						
Single	2,835 (32.0)	612 (45.3)	<0.001	1,309 (43.3)	508 (53.3)	<0.001
Married	5,620 (63.4)	681 (50.4)		1,576 (52.2)	382 (40.1)	
Unknown	405 (4.6)	57 (4.2)		135 (4.5)	63 (6.6)	

HPV, human papillomavirus; IQR, interquartile range, HPV positive: 16.0% clinical, 7.5% pathologic; HPV negative: 13.7% clinical, 4.5% pathologic; the rest based on best available information as determined by SEER. ^†^HPV positive: 16.8% clinical, 6.7% pathologic; HPV negative: 14.4% clinical, 3.8% pathologic; the rest based on best available information as determined by SEER.

**Table 2:** Recommended treatment by stage, HPV status, and sex.

Recommended treatment	HPV positive			HPV negative		
	Men *N* = 8,860	Women *N* = 1,350	*p* value	Men *N* = 3,020	Women *N* = 953	*p* value
All stages, N (%)						
No	1,640 (18.5)	305 (22.6)	0.002	538 (17.8)	202 (21.2)	0.06
Yes	7,198 (81.2)	1,042 (77.2)		2,474 (81.9)	748 (78.5)	
Missing	22 (0.3)	3 (0.2)		8 (0.3)	3 (0.3)	

Stage I, N (%)	*N* = 5,098	*N* = 878	0.02	*N* = 214	*N* = 121	0.18
No	1,071 (21.0)	220 (25.1)		17 (7.9)	5 (4.1)	
Yes	4,013 (78.7)	655 (74.6)		197 (92.1)	116 (95.9)	
Missing	14 (0.3)	3 (0.3)		0 (0.0)	0 (0.0)	

Stage II, N (%)	*N* = 2,097	*N* = 255	0.30	*N* = 250	*N* = 101	0.59
No	240 (11.4)	37 (14.5)		20 (8.0)	5 (5.0)	
Yes	1,854 (88.4)	218 (85.5)		227 (90.8)	95 (94.1)	
Missing	3 (0.1)	0 (0.0)		3 (1.2)	1 (1.0)	

Stage III, N (%)	*N* = 1,451	*N* = 181	0.70	*N* = 563	*N* = 170	0.03
No	247 (17.0)	34 (18.8)		64 (11.4)	32 (18.8)	
Yes	1,201 (82.8)	147 (81.2)		498 (88.5)	137 (80.6)	
Missing	3 (0.2)	0 (0.0)		1 (0.2)	1 (0.6)	

Stage IV (A/B/C), N (%)	*N* = 214	*N* = 36	0.84	*N* = 1,993	*N* = 561	0.005
No	82 (38.3)	14 (38.9)		437 (21.9)	160 (28.5)	
Yes	130 (60.8)	22 (61.1)		1,552 (77.9)	400 (71.3)	
Missing	2 (0.9)	0 (0.0)		4 (0.2)	1 (0.2)	

Abbreviations: HPV, human papillomavirus; N, number; %, percentage of total.

**Table 3:** Adjusted Cox proportional hazard models for overall survival (OS) and head and neck cancer-specific survival (HNCSS) by sex^∗^.

	OS				HNCSS			
	Men median, mo	Women median, mo	HR (95% CI)	*p* value	Men median, mo	Women median, mo	HR (95% CI)	*p* value
HPV positive								
All stages	—	—	1.05 (0.90–1.22)	0.57	—	—	1.21 (0.98–1.49)	0.08
Rec Trt	—	—	1.06 (0.89–1.28)	0.51	—	—	1.18 (0.91–1.51)	0.21
Stage I	—	—	0.94 (0.73–1.21)	0.61	—	—	1.12 (0.78–1.60)	0.53
Rec Trt	—	—	0.94 (0.70–1.27)	0.70	—	—	1.09 (0.72–1.65)	0.69
Stage II	—	—	1.13 (0.83–1.56)	0.43	—	—	1.46 (0.97–2.20)	0.07
Rec Trt	—	—	0.99 (0.68–1.44)	0.97	—	—	1.17 (0.71–1.92)	0.55
Stage III	80	—	1.21 (0.90–1.62)	0.20	—	—	1.36 (0.93–2.01)	0.11
Rec Trt	—	—	1.35 (0.96–1.88)	0.08	—	—	1.50 (0.97–2.31)	0.07
Stage IV	20	23	0.95 (0.57–1.59)	0.86	—	35	0.91 (0.44–1.89)	0.81
Rec Trt	32	31	1.01 (0.48–2.11)	0.98	—	—	0.61 (0.18–2.06)	0.42

HPV negative								
All stages	61	42	1.15 (1.02–1.29)	0.02^∗^	—	—	1.17 (0.99–1.38)	0.06
Rec Trt	81	50	1.28 (1.11–1.47)	0.001^∗^	—	—	1.30 (1.07–1.59)	0.009^∗^
Stage I	76	62	1.45 (0.90–2.32)	0.13	—	—	1.11 (0.49–2.50)	0.80
Rec Trt	—	62	1.57 (0.95–2.60)	0.08	—	—	1.18 (0.49–2.82)	0.72
Stage II	74	62	1.15 (0.76–1.72)	0.51	—	—	1.31 (0.72–2.39)	0.37
Rec Trt	74	62	1.27 (0.83–1.94)	0.27	—	—	1.48 (0.80–2.74)	0.21
Stage III	—	51	1.13 (0.83–1.53)	0.45	—	—	0.97 (0.64–1.47)	0.88
Rec Trt	—	62	1.43 (1.01–2.01)	0.04^∗^	—	—	1.09 (0.67–1.80)	0.72
Stage IVA	69	38	1.20 (1.01–1.43)	0.04^∗^	—	—	1.27 (1.00–1.61)	0.05^∗^
Rec Trt	81	47	1.22 (0.99–1.50)	0.06	—	—	1.38 (1.05–1.83)	0.02^∗^
Stage IVB	28	11	1.33 (0.93–1.91)	0.12	—	36	2.02 (1.26–3.24)	0.003^∗^
Rec Trt	48	18	1.72 (1.05–2.82)	0.03^∗^	—	41	2.64 (1.40–4.98)	0.003^∗^
Stage IVC	10	10	0.95 (0.63–1.43)	0.80	18	44	0.69 (0.39–1.21)	0.20
Rec Trt	11	16	1.09 (0.52–2.30)	0.81	25	—	0.90 (0.29–2.78)	0.86

^*∗*^Median survival not calculable for missing values. OS, overall survival; HNCSS, head and neck cancer-specific survival; HPV, human papillomavirus; HR, hazard ratio; CI, confidence interval; Rec Trt, recommended treatment.

## Data Availability

We used data from the Surveillance, Epidemiology, and End Results (SEER) Head and Neck with HPV Status Database, a national cancer registry that collects clinicopathologic data on incident cancer cases from 18 population-based registries. Access to data can be requested here: https://seer.cancer.gov/seerstat/databases/hpv/

## Supplementary Materials

Supplementary Materials A PDF containing three tables that have the complete multivariable models for overall survival and head and neck cancer-specific survival for HPV-positive cases (Supplementary Table 1) and HPV-negative cases (Supplementary Table 2), and treatment breakdown by stage, HPV status, and sex (Supplementary Table 3) is included as a Supplementary File.
