# Supplementary material for: Sex-Related Differences in Outcomes for Oropharyngeal Squamous Cell Carcinoma by HPV Status
Source: Int J Otolaryngol. 2022 May 2;2022:4220434. doi: 10.1155/2022/4220434 (PMC9085342; doi:10.1155/2022/4220434)
Supplement: Supplementary Materials — A PDF containing three tables that have the complete multivariable models for overall survival and head and neck cancer-specific survival for HPV-positive cases (Supplementary Table 1) and HPV-negative cases (Supplementary Table 2), and treatment breakdown by stage, HPV status, and sex (Supplementary Table 3) is included as a Supplementary File. [file 4220434.f1.doc]

| **Supplemental Table 1.** Multivariable model for overall survival (OS) and head and neck cancer-specific survival (HNCSS) for HPV positive cases | | | | | |
| --- | --- | --- | --- | --- | --- |
|  | | **OS** | | **HNCSS** | |
| **Category** | **Variable** | **HR (95% CI)** | ***p*-value** | **HR (95% CI)** | ***p*-value** |
| *Sex* | Male | Ref |  | Ref |  |
|  | Female | 1.05 (0.90-1.22) | 0.57 | 1.21 (0.98-1.49) | 0.08 |
| *Race* | White | Ref |  | Ref |  |
|  | Black | 1.32 (1.09-1.61) | 0.006 | 1.30 (0.98-1.73) | 0.07 |
|  | Hispanic | 1.03 (0.82-1.29) | 0.23 | 0.98 (0.71-1.36) | 0.91 |
|  | Asian or PI | 0.93 (0.63-1.37) | 0.71 | 0.87 (0.50-1.51) | 0.61 |
|  | Other/Unknown | 0.59 (0.27-1.32) | <0.001 | 0.98 (0.41-2.37) | 0.97 |
| *Age* | Age | 1.26 (1.22-1.29) | <0.001 | 1.21 (1.16-1.26) | <0.001 |
| *Clinical Stage* | I | Ref |  | Ref |  |
|  | II | 1.99 (1.73-2.30) | <0.001 | 2.21 (1.80-2.71) | <0.001 |
|  | III | 3.05 (2.65-3.50) | <0.001 | 3.63 (2.98-4.43) | <0.001 |
|  | IV | 6.68 (5.39-8.26) | <0.001 | 8.61 (6.43-11.53) | <0.001 |
| *Treatment* | No treatment | Ref |  | Ref |  |
|  | Surgery only | 0.21 (0.16-0.28) | <0.001 | 0.18 (0.12-0.28) | <0.001 |
|  | RT only | 0.23 (0.18-0.29) | <0.001 | 0.21 (0.15-0.30) | <0.001 |
|  | Chemo only | 0.38 (0.29-0.50) | <0.001 | 0.32 (0.22-0.47) | <0.001 |
|  | Surgery & RT | 0.12 (0.09-0.16) | <0.001 | 0.10 (0.06-0.15) | <0.001 |
|  | RT & chemo | 0.16 (0.14-0.20) | <0.001 | 0.16 (0.12-0.21) | <0.001 |
|  | Surgery & chemo | 0.33 (0.20-0.56) | <0.001 | 0.22 (0.10-0.52) | <0.001 |
|  | Surgery & RT & chemo | 0.12 (0.10-0.15) | <0.001 | 0.11 (0.08-0.16) | <0.001 |
| *Median Household Income* | Median Household Income | 1.00 (1.00-1.00) | 0.84 | 1.00 (1.00-1.00) | 0.87 |
| *% Highest Education Attained* | 9th grade | 1.00 (1.00-1.00) | 0.08 | 1.00 (1.00-1.00) | 0.75 |
|  | High school | 1.00 (1.00-1.00) | 0.07 | 1.00 (1.00-1.00) | 0.72 |
|  | At least bachelors | 1.00 (1.00-1.00) | 0.09 | 1.00 (1.00-1.00) | 0.03 |
| *Insurance* | Uninsured | Ref |  | Ref |  |
|  | Any Medicaid | 1.15 (0.86-1.54) | 0.35 | 1.27 (0.82-1.98) | 0.28 |
|  | Insured | 0.55 (0.41-0.72) | <0.001 | 0.68 (0.45-1.03) | 0.07 |
|  | Insured/No specifics | 0.69 (0.51-0.94) | 0.02 | 0.82 (0.52-1.28) | 0.38 |
|  | Unknown | 0.60 (0.35-1.04) | 0.07 | 0.85 (0.40-1.78) | 0.66 |

*Abbreviations: OS, overall survival; HNCSS, head and neck cancer-specific survival; HPV, human papillomavirus; HR, hazard ratio;*

*CI, confidence interval;* *RT, radiotherapy; Chemo, chemotherapy*

| **Supplemental Table 2.** Multivariable model for overall survival (OS) and head and neck cancer-specific survival (HNCSS) for HPV negative cases | | | | | |
| --- | --- | --- | --- | --- | --- |
|  | | **OS** | | **HNCSS** | |
| **Category** | **Variable** | **HR (95% CI)** | ***p*-value** | **HR (95% CI)** | ***p*-value** |
| *Sex* | Male | Ref |  | Ref |  |
|  | Female | 1.15 (1.02-1.29) | 0.02 | 1.17 (0.99-1.38) | 0.06 |
| *Race* | White | Ref |  | Ref |  |
|  | Black | 1.28 (1.10-1.48) | 0.001 | 1.36 (1.11-1.67) | 0.003 |
|  | Hispanic | 0.74 (0.59-0.94) | 0.01 | 0.76 (0.55-1.04) | 0.09 |
|  | Asian or PI | 0.92 (0.68-1.21) | 0.51 | 1.05 (0.72-1.51) | 0.81 |
|  | Other/Unknown | 1.62 (0.93-2.81) | 0.09 | 1.15 (0.48-2.80) | 0.75 |
| *Age* | Age | 1.15 (1.12-1.18) | <0.001 | 1.13 (1.09-1.18) | <0.001 |
| *Clinical Stage* | I | Ref |  | Ref |  |
|  | II | 1.57 (1.18-2.09) | 0.002 | 2.14 (1.36-3.38) | 0.001 |
|  | III | 1.72 (1.31-2.25) | <0.001 | 2.90 (1.89-4.43) | <0.001 |
|  | IVA | 2.17 (1.69-2.80) | <0.001 | 3.47 (2.30-5.23) | <0.001 |
|  | IVB | 3.43 (2.58-4.56) | <0.001 | 4.82 (3.06-7.59) | <0.001 |
|  | IVC | 4.96 (3.68-6.68) | <0.001 | 7.96 (5.03-12.60) | <0.001 |
| *Treatment* | No treatment | Ref |  | Ref |  |
|  | Surgery only | 0.31 (0.24-0.39) | <0.001 | 0.31 (0.23-0.43) | <0.001 |
|  | RT only | 0.37 (0.30-0.45) | <0.001 | 0.31 (0.23-0.41) | <0.001 |
|  | Chemo only | 0.51 (0.40-0.65) | <0.001 | 0.43 (0.31-0.60) | <0.001 |
|  | Surgery & RT | 0.14 (0.10-0.19) | <0.001 | 0.10 (0.06-0.15) | <0.001 |
|  | RT & chemo | 0.21 (0.18-0.25) | <0.001 | 0.19 (0.15-0.24) | <0.001 |
|  | Surgery & chemo | 0.24 (0.14-0.40) | <0.001 | 0.18 (0.08-0.38) | <0.001 |
|  | Surgery & RT & chemo | 0.15 (0.12-0.19) | <0.001 | 0.14 (0.10-0.19) | <0.001 |
| *Median Household Income* | Median Household Income | 1.00 (1.00-1.00) | 0.73 | 1.00 (1.00-1.00) | 0.81 |
| *% Highest Education Attained* | 9th grade | 0.99 (0.99-0.99) | 0.04 | 1.00 (1.00-1.00) | 0.70 |
|  | High school | 1.00 (1.00-1.00) | 0.07 | 1.00 (1.00-1.00) | 0.74 |
|  | At least bachelors | 1.00 (1.00-1.00) | 0.99 | 1.00 (1.00-1.00) | 0.21 |
| *Insurance* | Uninsured | Ref |  | Ref |  |
|  | Any Medicaid | 1.00 (0.79-1.27) | 0.98 | 1.13 (0.80-1.60) | 0.49 |
|  | Insured | 0.61 (0.48-0.76) | <0.001 | 0.74 (0.53-1.03) | 0.07 |
|  | Insured/No specifics | 0.76 (0.59-0.98) | 0.03 | 0.94 (0.65-1.35) | 0.74 |
|  | Unknown | 0.64 (0.41-0.98) | 0.04 | 0.62 (0.32-1.19) | 0.15 |

*Abbreviations: OS, overall survival; HNCSS, head and neck cancer-specific survival; HPV, human papillomavirus; HR, hazard ratio;*

*CI, confidence interval; RT, radiotherapy; Chemo, chemotherapy*

| **Supplemental Table 3.** Treatment breakdown by stage, HPV status, and sex | | | | | | |
| --- | --- | --- | --- | --- | --- | --- |
| **Treatment** | **HPV Positive** | | | **HPV Negative** | | |
|  | **Men**  **N=8,860** | **Women**  **N=1,350** | ***p*-value** | **Men**  **N=3,020** | **Women**  **N=953** | ***p*-value** |
| **All Stages**, N (%) |  |  | <0.001 |  |  | <0.001 |
| None | 275 (3.1) | 35 (2.6) |  | 199 (6.6) | 90 (9.4) |  |
| Surgery only | 595 (6.7) | 118 (8.7) |  | 310 (10.3) | 135 (14.2) |  |
| Radiation therapy only | 661 (7.5) | 128 (9.5) |  | 274 (9.1) | 115 (12.1) |  |
| Chemotherapy only | 176 (2.0) | 28 (2.1) |  | 118 (3.9) | 34 (3.6) |  |
| Surgery and radiation therapy | 947 (10.7) | 173 (12.8) |  | 221 (7.3) | 84 (8.8) |  |
| Radiation therapy and chemotherapy | 4,339 (49.0) | 566 (41.9) |  | 1,422 (47.1) | 355 (37.3) |  |
| Surgery and chemotherapy | 56 (0.6) | 9 (0.7) |  | 29 (1.0) | 10 (1.1) |  |
| Surgery, radiation therapy, and chemotherapy | 1,799 (20.3) | 293 (21.7) |  | 439 (14.5) | 127 (13.3) |  |
| Missing | 12 (0.1) | 0 (0.0) |  | 8 (0.3) | 3 (0.3) |  |
| **Stage I**, N (%) | N=5,098 | N=878 | 0.04 | N=214 | N=121 | 0.24 |
| None | 118 (2.3) | 13 (1.5) |  | 14 (6.5) | 4 (3.3) |  |
| Surgery only | 520 (10.2) | 106 (12.1) |  | 114 (53.3) | 70 (57.9) |  |
| Radiation therapy only | 382 (7.5) | 87 (9.9) |  | 27 (12.6) | 19 (15.7) |  |
| Chemotherapy only | 49 (1.0) | 7 (0.8) |  | 3 (1.4) | 1 (0.8) |  |
| Surgery and radiation therapy | 802 (15.7) | 149 (17.0) |  | 37 (17.3) | 18 (14.9) |  |
| Radiation therapy and chemotherapy | 1,933 (37.9) | 292 (33.3) |  | 6 (2.8) | 7 (5.8) |  |
| Surgery and chemotherapy | 32 (0.6) | 6 (0.7) |  | 0 (0.0) | 0 (0.0) |  |
| Surgery, radiation therapy, and chemotherapy | 1,258 (24.7) | 218 (24.8) |  | 13 (6.1) | 2 (1.7) |  |
| Missing | 4 (0.1) | 0 (0.0) |  | 0 (0.0) | 0 (0.0) |  |
| **Stage II**, N (%) | N=2,097 | N=255 | 0.14 | N=250 | N=101 | 0.82 |
| None | 58 (2.8) | 8 (3.1) |  | 17 (6.8) | 5 (5.0) |  |
| Surgery only | 48 (2.3) | 9 (3.5) |  | 68 (27.2) | 25 (24.8) |  |
| Radiation therapy only | 139 (6.6) | 20 (7.8) |  | 50 (20.0) | 27 (26.7) |  |
| Chemotherapy only | 40 (1.9) | 9 (3.5) |  | 3 (1.2) | 0 (0.0) |  |
| Surgery and radiation therapy | 95 (4.5) | 16 (6.3) |  | 42 (16.8) | 14 (13.9) |  |
| Radiation therapy and chemotherapy | 1,393 (66.4) | 145 (56.9) |  | 43 (17.2) | 20 (19.8) |  |
| Surgery and chemotherapy | 13 (0.6) | 1 (0.4) |  | 5 (2.0) | 1 (1.0) |  |
| Surgery, radiation therapy, and chemotherapy | 308 (14.7) | 47 (18.4) |  | 19 (7.6) | 8 (7.9) |  |
| Missing | 3 (0.1) | 0 (0.0) |  | 3 (1.2) | 1 (1.0) |  |
| **Stage III**, N (%) | N=1,451 | N=181 | 0.99 | N=563 | N=170 | 0.17 |
| None | 74 (5.1) | 10 (5.5) |  | 32 (5.7) | 16 (9.4) |  |
| Surgery only | 21 (1.5) | 3 (1.7) |  | 57 (10.1) | 21 (12.4) |  |
| Radiation therapy only | 123 (8.5) | 18 (9.9) |  | 42 (7.5) | 14 (8.2) |  |
| Chemotherapy only | 50 (3.5) | 6 (3.3) |  | 13 (2.3) | 6 (3.5) |  |
| Surgery and radiation therapy | 48 (3.3) | 6 (3.3) |  | 52 (9.2) | 18 (10.6) |  |
| Radiation therapy and chemotherapy | 917 (63.2) | 114 (63.0) |  | 270 (48.0) | 64 (37.7) |  |
| Surgery and chemotherapy | 7 (0.5) | 1 (0.6) |  | 4 (0.7) | 4 (2.4) |  |
| Surgery, radiation therapy, and chemotherapy | 208 (14.3) | 23 (12.7) |  | 92 (16.3) | 26 (15.3) |  |
| Missing | 3 (0.2) | 0 (0.0) |  | 1 (0.2) | 1 (0.6) |  |
| **Stage IV(A/B/C)**, N (%) | N=214 | N=36 | 0.67 | N=1,993 | N=561 | 0.004 |
| None | 25 (11.7) | 4 (11.1) |  | 136 (6.8) | 65 (11.6) |  |
| Surgery only | 6 (2.8) | 0 (0.0) |  | 71 (3.6) | 19 (3.4) |  |
| Radiation therapy only | 17 (7.9) | 3 (8.3) |  | 155 (7.8) | 55 (9.8) |  |
| Chemotherapy only | 37 (17.3) | 6 (16.7) |  | 99 (5.0) | 27 (4.8) |  |
| Surgery and radiation therapy | 2 (0.9) | 2 (5.6) |  | 90 (4.5) | 34 (6.1) |  |
| Radiation therapy and chemotherapy | 96 (44.9) | 15 (41.7) |  | 1,103 (55.3) | 264 (47.1) |  |
| Surgery and chemotherapy | 4 (1.9) | 1 (2.8) |  | 20 (1.0) | 5 (0.9) |  |
| Surgery, radiation therapy, and chemotherapy | 25 (11.7) | 5 (13.9) |  | 315 (15.8) | 91 (16.2) |  |
| Missing | 2 (0.9) | 0 (0.0) |  | 4 (0.2) | 1 (0.2) |  |

*Abbreviations: HPV, human papillomavirus; N, number; %, percentage of total*
